# The role of intra-articular injection of autologous platelet-rich plasma versus corticosteroids in the treatment of synovitis in lumbar facet joint disease

**DOI:** 10.15537/smj.2022.43.11.20220449

**Published:** 2022-11

**Authors:** Shahdan Y. Kotb, Nahed M. Sherif, Hala A. Saleh, Sahar F. Ahmed, Hossam M. Sakr, Mohamed O. Taeimah

**Affiliations:** *From the Department of Physical Medicine, Rheumatology and Rehabilitation (Kotb, Sherif, Saleh, Ahmed); from the Department of Radiodiagnosis (Sakr); and from the Department of Anesthesiology, Intensive Care and Pain Management (Taeimah), Faculty of Medicine, Ain Shams University, Cairo, Egypt.*

**Keywords:** platelet rich plasma, corticosteroids, synovitis, lumbar facet joint

## Abstract

**Objectives:**

To compare the efficacy between platelet-rich plasma (PRP) and corticosteroids (CS) in improving magnetic resonance imaging (MRI)-detected synovitis in correlation with clinical complaints among patients with lumbar facet joint (FJ) disease.

**Methods:**

This study was carried out at Eldemerdash Hospital, Cairo, Egypt between September 2019 and January 2021. A prospective, randomized, comparative, single blinded study included 30 patients with lumbar FJ disease, divided into 2 equal groups, received PRP and CS injections. Patients were comparatively assessed before and after the intervention according to number of tender lumbar FJs, maximum active lumbar extension range of motion, LBP visual analogue score, LBP functional disability questionnaires and MRI lumbar FJ detected synovitis and their grading.

**Results:**

Both groups showed a significant improvement in all mentioned parameters at follow-up after 3 months. However, PRP injections promoted better performance in terms of MRI synovitis grade in all lumbar FJ levels compared to CS injections.

**Conclusion:**

Both PRP and CS injections were effective in improving MRI-detected FJ synovitis while concurrently improving all examined parameters at follow-up after 3 months. However, PRP promoted better improvement in MRI-detected synovitis grade, suggesting that it may be a better treatment option for longer duration efficacy.

TRN: NCT04860531- 1/3/2021

**L**ow back pain (LBP) is a very common clinical condition occurring in 40%-85% of people at some point in their lives and whose impact extends from the individual to societal level.^1^

Determining the source of LBP is complex and studies have demonstrated that it mainly includes intervertebral discs, facet joints (FJs) and the sacroiliac joints.^2^ Lumbar disc herniation has been considered the leading cause of LBP. However, recent studies have shown that LBP is caused by lumbar FJ disease in nearly 45% of patients.^3^ Another study documented that up to 40% of a selected population who had LBP secondary to FJ disease and 27%-47% of those had false-positive findings, which comprised up to 80% of the studied group, showed pain relief using controlled local FJs anesthetic blocks.^4^

Magnetic resonance imaging (MRI) has been considered a safe imaging modality given its non-invasive and non-ionizing nature. It generates images with supreme soft tissue resolution while also allowing for the concurrent evaluation of the consequences of FJ degeneration, including active joint synovitis and effusion, adjacent and subchondral bone edema, neural structures impingement, and osteophytes formation.^5^ The MRI, specially fat saturated techniques, can accurately detect FJ degenerative synovitis using a grading system that appears to be well correlated with the patient’s pain.^6^

Treatment modalities of chronic LBP arising due to FJ disease include medial branch blocks, radiofrequency ablation neurotomy, and intra-articular injections. Lumbar FJ interventions can be useful for both the diagnosis and therapeutic management of chronic LBP, with the injections being performed through various techniques, although the fluoroscopy-guided technique has been considered the most accurate and reliable.^7^ Intra-articular FJ injection is frequently used as being proven beneficial in previous studies for facetogenic pain.^8^ Intra-articular steroid injections have shown various adverse effects including steroid-induced hyperglycemia, hypertension, gastrointestinal symptoms, exacerbation of heart failure, vertigo and urticaria.^9^ Therefore, identifying novel injectable drugs that are safe and efficacious for the treatment of lumbar FJ disease seems meaningful.^10^

Autologous PRP is prepared from the patient’s own blood, it is constituted from higher concentrations of platelets and different factors with potential healing abilities, that are delivered through alpha and delta granules. Among these factors; transforming growth factor beta and vascular endothelial growth factor which have a role in tissue regeneration and repair.^11^

Platelet-rich plasma (PRP) has the advantages of being simple to obtain and prepare, inexpressive, minimally invasive, and autologous in nature. Hence, PRP does not exhibit adverse effects typically present in other commonly used drugs. This has therefore encouraged several physicians to incorporate PRP into their practice as an alternative option to other more traditionally used intra-articular injectants, such as corticosteroids (CS) and hyaluronic acids.^12^

This study sought to compare the efficacy between intra-articular injections of PRP and CS in improving MRI-detected synovitis in correlation with clinical complaints among patients with lumbar FJ disease.

## Methods

The study was carried out at Eldemerdash Hospital, Ain Shams University, Cairo, Egypt between September 2019 and January 2021. After obtaining approval from the Ethics Committees of the Department of Physical Medicine, Rehabilitation and Rheumatology and the Faculty of Medicine, Ain Shams University, a prospective, randomized, comparative, single blinded study was carried out on 30 patients complaining of chronic LBP and suspected to have lumbar FJ disease who visited the outpatient clinic of Ain Shams University Hospital.

The current study included 30 patients based on a similar study by Wu et al^13^ who found that the objective success rate with over 50% pain relief at rest 3 month after treatment was 80% in the first group (PRP) and 15% in the second group (CS). Assuming power=0.80 and α=0.05 and using the 11th release of the Power Analysis and Sample Size software, version 11.0.3 (NCSS, Utah, USA), the minimal sample size for an assumed success rate of 50% was determined to be 30 cases (15 in each group).

We included participants between 20 and 40 years old who i) had continuous or intermittent gradually progressive back pain for more than 3 months, ii) showed no significant improvement despite different medical treatments (such as NSAIDs, gabapentinoids, and so on) or physical modalities, iii) had local/paraspinal pain or tenderness with or without radiation to buttocks, groin, or thigh, iv) had increased pain on extension, rotation, or lateral bending, and v) MRI-detected synovitis of the FJ as defined and graded by Czervionke and Fenton.^6^

Patients with acute low back trauma, fractures, malignancies, and inflammatory diseases; pain score of <4 at rest on a visual analog scale; radicular neurologic complaints; x-ray findings of chronic FJ disease indicated by marked joint space narrowing; subchondral sclerosis or bone deformity/spondylolisthesis; evident disc herniation on MRI; prior spinal surgery; prior interventional treatment on lumbar FJs; known hypersensitivity to local anesthesia, corticosteroids, contrast medium, or blood derivatives; local or systemic infection or spinal infection; uncorrectable coagulopathy; and diabetes mellitus, as well as pregnant women, were excluded from this study.

This study was carried out according to Helsinki declaration. After obtaining written informed consent, the recruited patients were subjected to the following: History taking and thorough clinical examination to exclude radicular or neurological manifestations (motor weakness, incontinence, and so on), detecting and documenting the number of FJs showing tenderness on palpation, and measuring the maximum active lumbar extension range of motion (ROM) using a goniometer for comparative purposes during follow-up.

Pain was analyzed using LBP visual analogue scale (VAS) at rest where 0 indicates no pain and 10 indicates the worst pain ever experienced. The Roland Morris Disability Questionnaire (RMQ) was used to determine patient disability within the last 24 hours. This tool has a total score of 24, with a score of 0 representing no disability and a score of 24 representing maximum disability.^14^

The Oswestry Disability Index (ODI) has been considered as the gold standard for measuring degree of disability and estimating quality of life in a patient with LBP.^13^ The ODI comprises 10 items that reflect the patient’s ability to manage their everyday life while dealing with their pain. A percentage score is calculated as follows: total score of the patient/max score possible × 100%. Accordingly, a 10% change has been identified as being clinically meaningful.^14^

This study utilized closed 1.5-Tesla MRI devices, with findings subsequently interpreted by a single blinded consultant radiologist to determine the presence of FJ synovitis and categorize them according to the following 5 grades based on the grading system developed by Czervionke and Fenton:^6^ Grade 0: no signal abnormality; Grade 1: signal abnormality confined to the joint capsule; Grade 2: periarticular signal abnormality involving <50% of the joint perimeter; Grade 3: periarticular signal abnormality involving >50% of the joint perimeter; and Grade 4: similar to Grade 3 with the extension of signal abnormality into the intervertebral foramen, ligamentum flavum, pedicle, transverse process, or vertebral body.

The patients were randomly assigned for intra-articular injection to either Group I (n=15) to be injected with autologous PRP and Group II (n=15) to be injected with CS (a mixture of 0.5% lidocaine and 5 mg/mL of betamethasone). The treated segments were determined using clinical signs and MRI-detected FJ synovitis.

### Preparation of autologous PRP

Under sterile conditions, approximately 20 mL of peripheral venous blood was collected with the addition of sodium citrate to the test tube. Platelet-rich plasma was prepared based on the standard 2-step centrifugation method using an on-desk centrifugation device-Centurion Scientific C2 series (Centurion Scientific Ltd, UK).

### Lumbar FJ injection

Injections were performed under fluoroscopic guidance utilizing the C-arm device with the supervision and guidance of an experienced pain consultant. Standard antiseptics were applied on the skin, after which local anesthesia with 0.5% lidocaine was administered followed by the insertion of a 21-G spinal needle into the FJ space under fluoroscopic guidance. Thereafter, the targeted joint was injected with approximately 0.5 mL of autologous PRP for the first group and 0.5 to 1 mL of a mixture comprising 0.5% lidocaine and 5 mg/mL of betamethasone for the second group. All patients were followed up 3 months after the injection. None of the patients received anti-inflammatory treatment during the follow-up period.Clinical evaluation for FJ tenderness and maximum active lumber extension ROM measuring were performed, after which LBP VAS at rest, RMQ, and ODI were calculated. All patients underwent MRI for FJ synovitis detection and grading of injected joints.

### Statistical analysis

Collected data were coded, tabulated, and statistically analyzed using the Statistical Package for Social Sciences for Windows, version 17.0 (IBMCorp, Armonk, NY, USA). Descriptive statistics were presented as mean±SD (standard deviation) and minimum and maximum ranges for numerical parametric data, median and 1st and 3rd inter-quartile range for numerical non-parametric data, and numbers and percentages for categorical data. Inferential analyses were carried out for quantitative variables using the independent t-test for 2 independent groups with parametric data and Mann–Whitney U for 2 independent groups with non-parametric data. Inferential analyses for qualitative data were performed using the Chi-square test for independent groups. The level of significance was set at a *p*-value of <0.05.

## Results

The total number of patients included in the study were 16 females (53.3%) and 14 males (46.7%) (mean±SD age of 36.23±3.63 years; range 29 to 40 years). Their disease duration ranged from 3 to 12 months with a mean±SD of 7.40±3.08 months. No statistically significant difference (*p*>0.05) was observed between both study groups with regard to age, gender, and disease duration. Statistical comparative analysis of all documented parameters was performed for both groups prior to the intervention. No significant differences (*p*>0.05) in the number of tender lumbar FJs, maximum active lumbar extension ROM, LBP VAS score, and functional disability questionnaires (RMQ and ODI scores) were observed between the 2 groups at base line. Moreover, no significant differences (*p*>0.05) in the number of FJs showing synovitis on MRI imaging and average grading of the synovitis were observed between the 2 groups.

At follow-up after 3 months, clinical assessment of patients in Group I showed a significant decrease in the number of tender lumbar FJs on palpation (*p*<0.05) and the percentage of tender FJs, suggesting an improvement in most of the lumber levels (L2-3, 3-4, and 4-5) after intra-articular PRP injection.

A highly significant increase in the maximum active lumber extension ROM (*p*<0.01) and highly significant decrease (*p*<0.01) in the LBP VAS at rest, RMQ, and ODI scores were observed, suggesting improvement in all parameters (Table 1).

**Table 1:** - Comparative results in Group I (platelet-rich plasma injection).

Parameters	Before	After	Test value*	*P*-value	Sig.
	n=15	n=15			
***Ex. ROM***					
Range	15–34	19–34	4.363	0.001	HS
Mean ± SD	23.60 ± 5.37	26.53 ± 4.09			
***VAS***					
Range	7–9	4–7	−9.934	<0.001	HS
Mean ± SD	8.00 ± 0.76	5.73 ± 0.88			
***RMQ***					
Range	12–22	10–18	−10.717	<0.001	HS
Mean ± SD	19.33 ± 2.55	14.27 ± 2.37			
***ODI***					
Range	32–70	38–64	−4.979	<0.001	HS
Mean ± SD	58.13 ± 10.43	47.60 ± 7.06			

*p*>0.05: not significant (NS), *p*<0.05: significant (S), *p*<0.01: highly significant (HS), RMQ: Roland Morris Questionnaire, ODI: Oswestry Disability Index, Ex. ROM: extension range of motion, VAS: visual analogue scale, *: paired t-test

After comparing MRI findings of lumbar FJs showing synovitis before and after the intervention, we found a highly significant decrease in synovitis grade (*p*<0.01) at levels L2-3, 3-4, and 4-5, indicating improvement, and a significant decrease in synovitis grade (*p*<0.05) at levels L1-2 and L5-S1 (Table 2).

**Table 2:** - Comparison between MRI lumbar facet joints synovitis grading before and after the intervention in Group I receiving platelet-rich plasma at each lumbar level.

MRI	Before		After		Test value*	*P*-value	Sig.
	n	%	n	%			
***L1-2 (n=5 [16.7%])***							
Grade L1-2							
Grade 0	0	0.0	4	80.0	6.800	0.033	S
Grade I	4	80.0	1	20.0			
Grade II	1	20.0	0	0.0			
***L2-3 (n= 7 [76.7%])***							
Grade L2-3							
Grade 0	0	0.0	6	85.7	10.571	0.005	HS
Grade I	6	85.7	1	14.3			
Grade II	1	14.3	0	0.0			
Grade III	0	0.0	0	0.0			
***L3-4 (n=9 [70.0%])***							
Grade L3-4							
Grade 0	0	0.0	7	77.8	11.455	0. 001	HS
Grade I	9	100.0	2	22.2			
Grade II	0	0.0	0	0.0			
Grade III	0	0.0	0	0.0			
***L4-5 (n=11 [63.3%])***							
Grade L4-5							
Grade 0	0	0.0	7	63.6	11.600	0.009	HS
Grade I	8	72.7	2	18.2			
Grade II	2	18.2	2	18.2			
Grade III	1	9.1	0	0.0			
***L5-S1 (n=13 [56.7%])***							
Grade L5–S1							
Grade 0	0	0.0	6	46.2	8.000	0.018	S
Grade I	10	76.9	6	46.2			
Grade II	3	23.1	1	7.7			
Grade III	0	0.0	0	0.0			

*p*>0.05: not significant (NS), *p*<0.05: significant (S), *p*<0.01: highly significant (HS), *:Chi-square test, MRI: magnetic resonance imaging

Regarding the findings for Group II 3 months after the intervention, we also found a significant decrease (*p*<0.05) in the percentage of tender FJs (L2-3, 3-4, 4-5, and L5–S1), suggesting improvement.

Our findings also showed a highly significant increase (*p*<0.01) in the maximum active lumber extension ROM and highly significant decrease (*p*<0.01) in LBP VAS at rest, RMQ, and ODI scores, suggesting improvement (Table 3).

**Table 3:** - Comparative results in group II (Corticosteroids).

Parameters	Before	After	Test value*	*P*-value	Sig.
	n=15	n=15			
***Ex. ROM***					
Range	10–30	18–32	6.808	<0.001	HS
Mean ± SD	21.47 ± 6.58	25.53 ± 4.70			
***VAS***					
Range	7–9	3–7	−10.044	<0.001	HS
Mean ± SD	8.07 ± 0.80	5.73 ± 1.39			
***RMQ***					
Range	12–22	10–19	−8.503	<0.001	HS
Mean ± SD	19.13 ± 2.45	15.20 ± 2.37			
***ODI***					
Range	32–72	28–60	−8.270	<0.001	HS
Mean ± SD	61.60 ± 10.62	50.13 ± 10.70			

*p*>0.05: not significant (NS); *p*<0.05: significant (S); *p*<0.01: highly significant (HS); RMQ: Roland Morris Questionnaire; ODI: Oswestry Disability Index; Ex. ROM: Extension range of motion; VAS: visual analogue scale; •: paired t-test

A comparison of MRI findings on lumbar FJs showing synovitis before and after the intervention in Group II revealed a highly significant decrease in synovitis grade (*p*<0.01) at level L5–S1, suggesting improvement, and a significant decrease (*p*<0.05) in synovitis grade at level L4-5. Meanwhile, no significant difference (*p*>0.05) was observed between the synovitis grade at the rest of the levels (Table 4).

**Table 4:** - Comparison of lumbar facet joint synovitis grading on MRI before and after the intervention in Group II receiving corticosteroids.

MRI	Before		After		Test value*	*P*-value	Sig.
	n	%	n	%			
***L1–2 (n=2 (6.7%))***							
Grade L1–2							
Grade 0	0	0.0	1	50.0	1.333	0.248	NS
Grade I	2	100.0	1	50.0			
Grade II	0	0.0	0	0.0			
Grade III	0	0.0	0	0.0			
***L2–3 (n=1 (3.3%))***							
Grade L2–3							
Grade 0	0	0.0	1	100.0	2.000	0.157	NS
Grade I	1	100.0	0	0.0			
Grade II	0	0.0	0	0.0			
Grade III	0	0.0	0	0.0			
***L3–4 (n=7 (23.3%))***							
Grade L3–4							
Grade 0	0	0.0	3	42.9	5.619	0.132	NS
Grade I	5	71.4	2	28.6			
Grade II	1	14.3	2	28.6			
Grade III	1	14.3	0	0.0			
***L4–5 (n=13 (43.3%))***							
Grade L4–5							
Grade 0	0	0.0	3	23.1	9.714	0.021	S
Grade I	5	38.5	9	69.2			
Grade II	6	46.2	1	7.7			
Grade III	2	15.4	0	0.0			
***L5–S1 (n=13 (46.7%))***							
Grade L5–S1							
Grade 0	0	0.0	7	53.8	11.328	0.001	HS
Grade I	7	53.8	6	46.1			
Grade II	6	46.1	0	0.0			
Grade III	0	0.0	0	0.0			

*p*>0.05: not significant (NS), *p*<0.05: significant (S), *p*<0.01: highly significant (HS), *: Chi-square test, MRI: magnetic resonance imaging

Comparative analysis of all documented parameters was also performed for both groups after the intervention. Accordingly, no significant differences (*p*>0.05) in the number of FJs showing tenderness on palpation and maximum active lumbar extension ROM were observed between the 2 groups. Similarly, no significant differences (*p*>0.05) in the LBP VAS and RMQ, and ODI scores were observed between both groups.

No significant difference (*p*>0.05) in FJs with synovitis was observed between both groups 3 months after the intervention. However, as mentioned before, joints injected with PRP showed a significantly greater decrease in synovitis grades on MRI at all lumbar FJ levels, suggesting improvement compared to those injected with CS, which showed a significant decrease in synovitis grade at only 2 levels.

## Discussion

Low back pain is one of the most common musculoskeletal conditions, it is considered to be a global health concern with an estimated lifetime prevalence of 50%-80%. Therefore, LBP is a leading cause of disability and work absenteeism resulting in a considerable economic and social burdens.^15^

The current study aimed to evaluate whether PRP could be an effective intra-articular injectant by comparing it to CS based on their ability to improve FJ with synovitis detected using MRI in correlation with clinical findings and LBP functional disability questionnaire results. This study compared findings before the intervention to those at follow-up 3 months after the intervention.

In out study, there was no significant difference (*p*>0.05) in age, gender, and disease duration between both study groups. Before the intervention, no significant difference (*p*>0.05) was observed between the 2 groups regarding the number of tender lumbar FJs, maximum active lumbar extension ROM, LBP VAS score, functional disability questionnaires (RMQ and ODI scores), number of lumbar FJs showing synovitis on MRI, and average synovitis grading.

After PRP injection in Group I, our findings showed a clinical improvement denoted by a significant (*p*<0.05) decrease in the number of lumbar FJs showing tenderness on palpation. This could be attributed to the healing ability of PRP as suggested by the study of Rothenberg et al,^16^ which showed that PRP participates in the healing process by delivering growth factors and other active molecules, contributing to diverse roles such as proliferation, angiogenesis, vessel remodeling, coagulation, and cell differentiation, all of which promote tissue healing and repair.

Moreover, our results for Group I showed a highly significant decrease (*p*<0.01) in mean VAS scores after the injection, which was associated with a concurrent highly significant increase in the active lumbar extension ROM. These results are consistent with those presented in Kumar et al^17^ who also detected the same significant results regarding VAS score and ROM upon using PRP as an injectant in knee osteoarthritis (OA).

Regarding the post-injection results of the functional disability questionnaires (RMQ and ODI) in Group I, we found a highly significant (*p*<0.01) decrease in both scores, suggesting marked functional improvement. This could be explained by the previously mentioned results of improvement in both the VAS score and lumber ROM, which in turn improved the functional abilities of the patients. These results are in agreement with those of Wu et al^10^ who also reported an improvement in functional capacity using the same questionnaires after intra-articular lumbar FJ injection with PRP.

The post-injection MRI results for the lumbar FJs in Group I showed a significant decrease in the number of joints exhibiting signs of synovitis and a decrease in synovitis grading at all lumbar FJ levels. This could be explained by the tissue healing and repair abilities of the PRP as mentioned earlier. To the best of our knowledge, no other study has used this objective method of detecting improvement in FJ synovitis on MRI in correlation with clinical findings and functional disability questionnaires. However, various studies have shown a strong correlation between the presence of synovitis on MRI and joint pain. Indeed, a study by Czervionke and Fenton^6^ on lumbar FJ synovitis carried out that the side of the facet synovitis detected on MRI correlated well with the side at which the patient complained of pain. Similarly, a study by Ahedi et al^18^ on the hip joint found a significant correlation between the amount of effusion and degree of pain reported by the patients.

The improvement in lumbar FJ synovitis, which is a sign of inflammation, after intra-articular injection with PRP in Group I can be attributed to the fact that PRP is a natural source of signaling molecules that aid in the resolution of inflammation. This finding is in agreement with that reported by Xu et al^19^ who stated that upon activation of platelets in PRP, a release multiple growth factors and cytokines, that are involved in tissue regeneration and repair, among which; platelet-derived growth factor (PDGF), vascular endothelial growth factor (VEGF) and transforming growth factor-β (TGF-β) that are involved in promoting tissue repair and regeneration.

Our results for Group II showed a significant decrease in the number of lumbar FJs tender on palpation denoting clinical improvement after intra-articular injection with CS. This result could have been attributed to the well-known role of CS as a potent anti-inflammatory and analgesic. Choueiri et al^20^ also recently stated that CS has the potential to become the most commonly recommended intra-articular injection drug for the management of joint OA.

As suggested by Ferrara et al,^21^ the physiological effects of CS are initiated through the binding to the glucocorticoid receptor, which is characterized by being transcription factors: on ligand binding, they interact with specific DNA motifs that modulate transcription or repression of various genes expressed in immune cells, thus producing actions that are considered as anti-inflammatory and metabolic.

Furthermore, results for Group II after CS injection showed a significant decrease in pain score and consequently an increase in lumbar extension ROM, indicating improvement. This could have also been attributed to the anti-inflammatory nature of CS as mentioned previously. The decrease in pain scores after CS lumbar FJ injection had also been shown by Kwak et al^22^ who demonstrated a significant decrease in numerical rating scores at 1, 2, and 3 months after intra-articular lumbar FJ CS injection in 50 patients complaining of LBP due to FJ disease.

Additionally, our results for Group II showed a highly significant decrease in the functional disability questionnaires (RMQ and ODI), indicating functional improvement after CS injection. This functional improvement could have been a consequence of the decrease in pain scores and increased ROM. Our results regarding the functional disability questionnaires agrees with those reported by Wu et al^13^ who also showed a significant decrease in the same functional disability questionnaires scores (RMQ and ODI) after administering intra-articular CS to patients with lumbar FJ. Moreover, another study by Ragheb et al^23^ carried on 23 patients receiving lumbar FJ intra-articular CS injection reported a significant decrease in the ODI scores at the 3-month follow-up after the intervention, although our findings only partly agree with their results.

In our study, MRI findings of lumbar FJ synovitis in Group II after CS injection showed a significant decrease in the total number of joints showing synovitis and their grading; however, the decrease in synovitis grade was mainly significant at levels L4-5 and L5-S1. The lack of a statistically significant improvement at the rest of lumbar FJ levels could be attributed to the fact that L4–5 and L5–S1 were the most affected levels before the intervention in Group II. This can also be explained by the decrease in the anti-inflammatory effects of CS with time considering that our follow-up was conducted 3 months after CS injection. This explanation is supported by the findings of Annaswamy et al^24^ who carried out a clinical study utilizing intra-articular injection of triamcinolone in patients with lumbar FJ disease. Notably, the aforementioned study concluded that significant short-term functional benefit and pain decrease were present at the 1-month follow-up; however, no significant long-term functional and pain improvement had been observed at the 3- and 6-month follow-up.

The decrease in synovitis grading of lumbar FJs in Group II after the injection could be attributed to the known anti-inflammatory action of CS, as mentioned earlier. Our results partly agree with those of Riis et al^25^ who also determined synovitis grading on MRI before and after intra-articular injection with CS in patients with knee OA and reported significant improvement in correlation with clinical improvement.

The comparison between Groups I and II after injection showed no significant difference (*p*>0.05) regarding LBP VAS and RMQ and ODI scores. This finding is consistent with that reported by Wu et al^14^ who compared intra-articular injections of PRP and CS for the treatment of lumbar FJ disease in a prospective study carried on 46 patients. The mentioned study showed that both groups demonstrated statistically comparable improvements in VAS, RMQ, and ODI scores.

Our findings are in partial agreement with Ruiz‐Lopez and Tsai^26^ who also compared the efficacy and safety between fluoroscopically guided caudal epidural injections of PRP and CS in patients with complex chronic lumbar spinal pain. Accordingly, their results showed a significantly lower VAS score at the 1-month follow-up in patients who received CS injection. However, patients who received PRP had lower scores at the 3- and 6-month follow-up. Their study concluded that both PRP and CS were comparable in terms of safety and therapeutical effectiveness in patients with complex chronic lumbar spinal pain; however, PRP had been shown to be superior to CS for prolonged pain relief and improvement in quality of life.

The comparable results between both of our groups after the injection are consistent with a systematic review by Ling et al^27^ that included comparative studies on the clinical outcomes of PRP and CS injections for the treatment of lumbar spondylosis and sacroiliac arthropathy. Among the 5 studies (242 patients, among whom 114 and 128 received PRP and CS) analyzed, 4 found that both PRP and CS treatment showed a statistically comparable reduction in VAS scores of the patients, whereas the remaining study showed that only the PRP group exhibited a significant reduction in VAS scores. Three of the studies found that PRP patients had greater improvements in one or more of the clinical outcome scores compared to CS patients at the 3- to 6-month follow-up. The systematic review concluded that both PRP and CS injections were comparable in terms of safety and efficacy, with some evidence suggesting that PRP injection would be a more effective option for long-term follow-up compared to CS injection.

### Study limitations

The small sample size included in this study was due to the circumstances of COVID19 pandemic, therefore this study was carried out on the minimal accepted sample size as justified assuming power=0.80 and α=0.05 and using the 11th release of the PASS software, the minimal sample size for an assumed success rate of 50% was determined to be 30 cases (15 in each group) based on a similar study by Wu et al^13^ who found that the objective success rate with over 50% pain relief at rest 3 month after treatment was 80% in the first group (PRP) and 15% in the second group (CS).

In conclusion, intra-articular injections of both PRP and CS were determined to be effective for the treatment of MRI-detected FJ synovitis in correlation with improved clinical findings, pain, and functional activities at follow-up after 3 months. However, autologous PRP showed superior improvement in synovitis grading on MRI and may therefore be a better treatment option provided its prolonged efficacy and fewer side effects.
